# Identifying inhibitors of the *Leishmania* inositol phosphorylceramide synthase with antiprotozoal activity using a yeast-based assay and ultra-high throughput screening platform

**DOI:** 10.1038/s41598-018-22063-9

**Published:** 2018-03-02

**Authors:** Jennifer L. Norcliffe, John G. Mina, Emilio Alvarez, Juan Cantizani, Francisco de Dios-Anton, Gonzalo Colmenarejo, Silva Gonzalez-Del Valle, Maria Marco, José M. Fiandor, Julio J. Martin, Patrick G. Steel, Paul W. Denny

**Affiliations:** 1 Department of Biosciences, Durham University, Stockton Road, Durham, DH1 3LE UK; 2 Department of Chemistry, Durham University, Stockton Road, Durham, DH1 3LE UK; 3 GlaxoSmithKline Investigacion y Desarrollo, Parque Tecnologico de Madrid, 28760 Tres Cantos Madrid, Spain; 4 Biostatistics and Bioinformatics Unit, IMDEA Food Institute, CEI UAM and CSIC, Carretera de Cantoblanco 8, 28049 Madrid, Spain

## Abstract

Leishmaniasis is a Neglected Tropical Disease caused by the insect-vector borne protozoan parasite, *Leishmania* species. Infection affects millions of the world’s poorest, however vaccines are absent and drug therapy limited. Recently, public-private partnerships have developed to identify new modes of controlling leishmaniasis. Drug discovery is a significant part of these efforts and here we describe the development and utilization of a novel assay to identify antiprotozoal inhibitors of the *Leishmania* enzyme, inositol phosphorylceramide (IPC) synthase. IPC synthase is a membrane-bound protein with multiple transmembrane domains, meaning that a conventional *in vitro* assay using purified protein in solution is highly challenging. Therefore, we utilized *Saccharomyces cerevisiae* as a vehicle to facilitate ultra-high throughput screening of 1.8 million compounds. Antileishmanial benzazepanes were identified and shown to inhibit the enzyme at nanomolar concentrations. Further chemistry produced a benzazepane that demonstrated potent and specific inhibition of IPC synthase in the *Leishmania* cell.

## Introduction

The Neglected Tropical Disease (NTD) leishmaniasis is endemic in over 90 countries worldwide, affecting approximately 12 million people per year with 350 million people living at risk of disease. The causative agent, *Leishmania* species, are sandfly borne kinetoplastid protozoan parasites^1^.

A vaccine to prevent leishmaniasis is not currently available and treatment relies entirely on a limited number of chemotherapeutics with, in the most part, unclear modes of action^2^. For example, cutaneous leishmaniasis (CL, e.g. caused by *L*. *major*) therapy largely relies on two pentavalent antimonials, sodium stibogluconate (Pentostam) and meglumine antimoniate (Glucantime)^3,4^. Despite their problems, including severe side-effects such as cardiotoxicity and the requirement for parenteral administration^5^, Pentostam and Glucantime have been in clinical use for more than 70 years^6^. The treatment options for visceral leishmaniasis (VL, Kala-Azar; largely caused by *L*. *donovani*) suffer from similar problems, with antimonial therapy still widely employed^3,4^. In addition, resistance to these drugs is rising and threatening their continued use in the treatment of leishmaniasis^7^. Amphotericin B (Fungizone) has also been used in the treatment of both CL and VL^8^. However, this is also associated with severe side-effects, and must be administered parentally^9^. Miltefosine, is the only orally available antileishmanial and has been used to treat VL in India since 2002, however, this antineoplastic compound also exhibits teratogenicity and resistance is increasingly apparent^10^. Therefore, the current elimination programme in South Asia relies upon liposomal amphotericin B administered as a single injection. However, whilst effective and less toxic than the non-liposomal formulation, treatment failure and post-Kala-Azar dermal leishmaniasis (PKDL) may preclude elimination^11^.

With these severe problems in the treatment and control of both CL and VL, it is recognized that new antileishmanial targets and drugs need to be identified. High throughput screening (HTS) is a vital component of drug discovery and can be carried out using either *in vitro* assays against validated targets or in phenotypic assays against the parasite itself^12,13^. Recent high content phenotypic screening across the pathogenic kinetoplastids gave a disappointingly low number of novel potent hits against *Leishmania donovani* when compared with the related parasites *Trypanosoma brucei* and *T*. *cruzi*^14^. This demonstrated the importance of target-based screening for antileishmanial discovery to access to novel chemical space and new modes of action which may overcome resistance and be compatible in combination therapies.

In contrast to mammalian cells, where the predominant complex sphingolipid is sphingomyelin (SM), fungi, plants and some protozoa synthesize inositol phosphorylceramide (IPC)^15,16^. The fungal IPC synthase (AUR1p) has long been established as a drug target for pathogenic fungi^17^. Similarly, the *Leishmania* orthologue, and those from the pathogenic *Trypanosoma* species, have been suggested to be ideal, non-mammalian, targets for the development of new, less toxic, antiprotozoals^15,18–20^. Furthermore, systems biology studies have reinforced the status of the *Leishmania* enzyme as a putative target for drug discovery programmes^21^. However, as an integral membrane protein with 6 transmembrane domains, and lipid substrates (phosphatidylinositol and ceramide) and products (diacylglycerol and IPC), formatting the *Leishmania* IPC synthase into a conventional *in vitro* assay platform is challenging^22^. Therefore, utilizing the ability of the kinetoplastid enzyme to complement for the absence of the *Saccharomyces cerevisiae* orthologue AUR1p^23^, here we describe the development and formatting of a robust yeast-based ultra-HTS (uHTS) assay platform. This was then utilized, in the largest effort of its type, to screen a high content (1.8 M) compound library for specific *Leishmania* IPC synthase inhibitors. 500 potent and specific such compounds were identified; these were then reduced to 211 following clustering to remove structural replicates. Following screening against mammalian-stage axenic amastigote *L*. *donovani*, 25 of these compounds were selected on the basis of potency, selectivity and physicochemical properties. These hits were then reduced to five following further screening of *L*. *donovani* infected macrophages. From these, one pair of structurally related compounds, the benzazepanes, was selected for further analyses. Importantly, these compounds demonstrated sub micro-molar activity against the enzyme target in a secondary *in vitro* assay and selectivity for the enzyme *in cellulo* when using an available *L*. *major* sphingolipid mutant^24^. This work demonstrated the tractability of yeast-vehicles for uHTS, with the identification of the benzazepanes as potential antileishmanials with specific inhibitory activity against IPC synthase.

## Results

### Design and validation of a robust *Leishmania* IPC synthase assay for uHTS

HTS campaigns predominantly rely on two approaches: (i) *in vitro* target-directed screening using biochemical assays; (ii) cell-based phenotypic screening which takes no account of the target. Both of these approaches have significant limitations, such as the production of soluble protein and a lack of cellular context for biochemical assays, and the problems of process redundancy in cells used for phenotypic screening^13^. Furthermore, both can be difficult to miniaturize and expensive to utilize for uHTS. With these problems in mind, yeast-based systems have been utilized to provide cost-effective, target-directed, screening platforms for protein targets within a eukaryotic cellular context^13^. Recently, this approach has been adopted for antiprotozoal drug discovery^12,25,26^.

**Figure 1:**
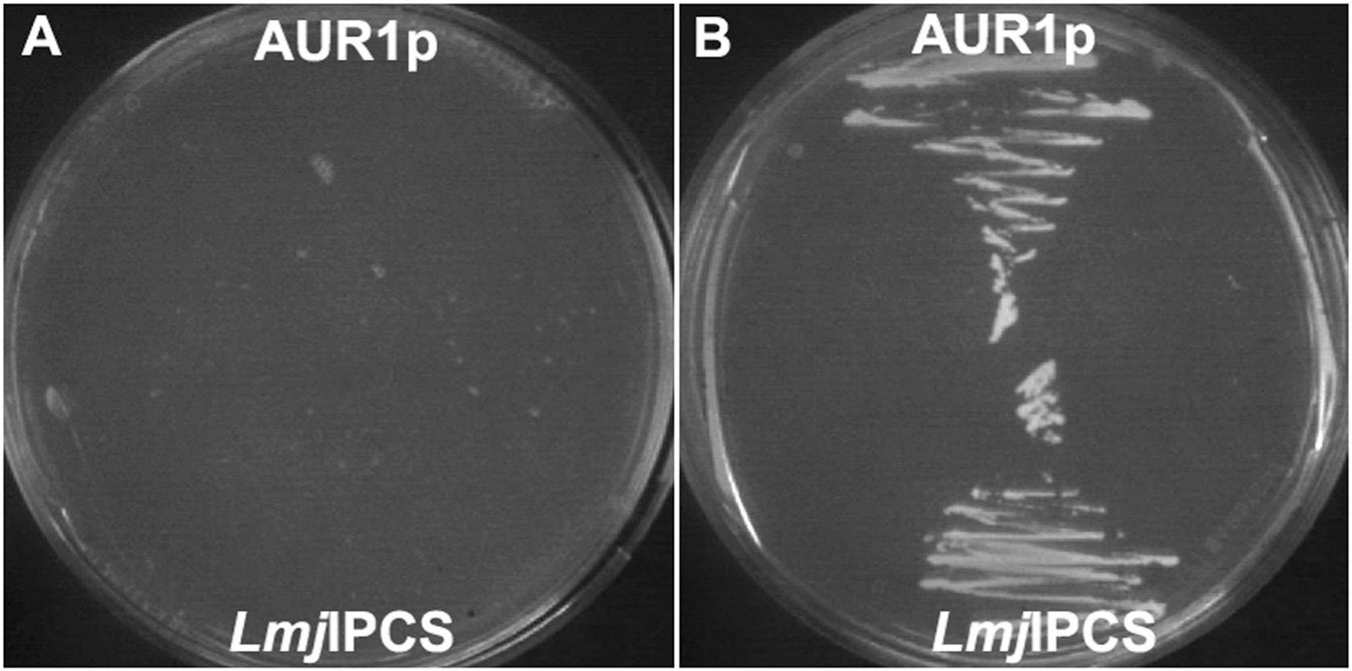
Generation of yeast strains for uHTS assay. Yeast lacking AUR1p completed by galactose dependent expression of AUR1p (*α ade*^*−*^.*lys*^*−*^.*leu*^*−*^.*Δaur1*^*−*^.pESC-LEU_AUR1) or *LmjF*IPCS (*α ade*^*−*^.*lys*^*−*^.*leu*^*−*^.*Δaur1*^*−*^.pESC-LEU_*LmjF*IPCS) grown on selective media with either glucose (**A**) or galactose (**B**). Expression of both AUR1p and *LmjF*IPCS rescues growth of the mutant *S*. *cerevisiae*.

The transmembrane nature of the target *Leishmania* IPC synthase, and the hydrophobicity of the lipid substrates and products, rendered it challenging to develop a uHTS biochemical assay^18,19,22^. However, given that the enzyme has been shown to complement an auxotrophic *Saccharomyces cerevisiae* mutant^18^ the development of yeast-based screening platform was considered tractable. To develop a robust assay suitable for uHTS, rather than the previously utilized auxotrophic mutant^18^, a strain of *S*. *cerevisiae* completely lacking the yeast IPC synthase, AUR1p, was selected as the vehicle (a kind gift from Teresa Dunn, Uniformed Services University)^27^. This strain is reliant on the expression of essential AUR1p from a uracil selectable expression plasmid (pRS316-URA-AUR1)^27^. Transforming these with the plasmid pESC-LEU (Agilent) harbouring the *L*. *major* IPC synthase (*LmjF*IPCS) or AUR1p under the control of a galactose promotor, allowed for curing the yeast of pRS316-URA-AUR1 using the pyrimidine analogue 5-fluoro-orotic acid (5-FOA) and the selection of a strain dependant on the galactose inducible expression of *LmjF*IPCS (Fig. 1). The yeast dependant on either *LmjF*IPCS or AUR1p expression were then used to format an *LmjF*IPCS assay suitable for uHTS.

Exploiting the fact that growing yeast secretes *exo-β-*glucanase, which, as well as modifying the cell wall, can also hydrolyse non-fluorescent fluorescein di-(*β*-D-glucopyranoside) (FDGlu) to release fluorescein, an assay was developed in which cell growth was monitored by fluorescent output^28^. The optimal concentrations of galactose (0.1%), starter culture (OD_600_ 0.063) and FDGlu (10 μM) were established in 96- and 384-well formats incubated at 30 °C for 24 hours. The assay was then miniaturized into a 1536-well format, the most appropriate positive control (cycloheximide) selected and the robustness of the platform indicated by Z’ values >0.5^29^. Screening using a GSK standard set of (9766) compounds further validated the assay, established inhibition threshold values and indicated a hit-rate of 1.07%.

### Identification of selective and potent inhibitors

Taking the robust 1536-well format assay developed, a primary uHTS screening was conducted using the 1.8 million compound GSK library against the *LmjF*IPCS dependent yeast. Given the hit-rate of 1.07% with the standard set approximately 19000 hits were anticipated. Initially this expectation was not reached, however reducing the inhibition threshold to capture small (≤300 Da molecular weight), polar (cLogP ≤ 3) ‘drug-like’ compounds with lower efficacy, led to the identification of 19669 hits. The mean Z’ for the screen was 0.70, demonstrating the platform to be robust.

At this stage, the primary screen, it was unclear whether the hit compounds inhibited the *LmjF*IPCS enzyme or (an)other essential process(es) within the yeast vehicle. Therefore, a counter screen was performed against the yeast dependant on AUR1p expression at a single compound concentration of 10 μM. In parallel, a confirmatory screen against the *LmjF*IPCS dependent yeast was performed at the same concentration. After calculation of the inhibition thresholds for each strain, those compounds demonstrating activity above the *LmjF*IPCS threshold but below AUR1p threshold were selected. In addition, to avoid loss of highly potent compounds, those demonstrating >80% inhibition of the *LmjF*IPCS dependent yeast were retained whatever the response against AUR1p dependent yeast.

**Figure 2:**
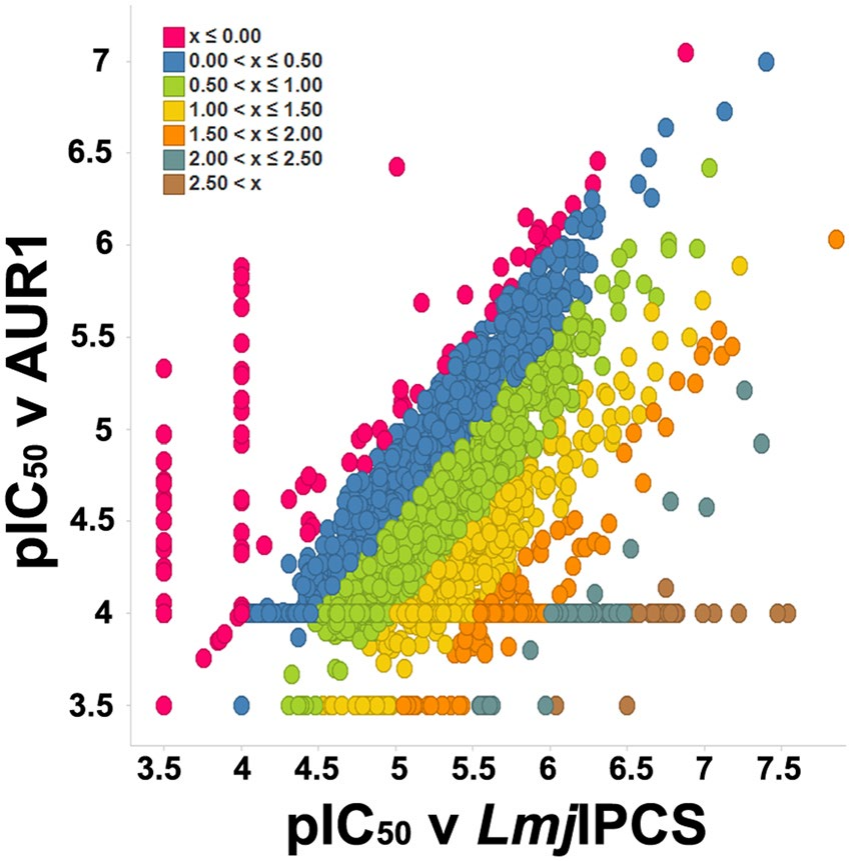
The dose response (−logIC_50_ M [pIC_50_]) of the initially identified 4166 selective hit compounds. Yeast dependent on *Leishmania* IPC synthase activity (*LmjF*IPCS) against yeast dependent on AUR1p expression. The inset shows the colour key for compound selectivity (logSI [pSI] where SI is Selectivity Index). 500 hits with a pSI > 1.50 were selected for further analyses (orange, turquoise and brown).

As a result of this process, 4166 compounds were progressed further for dose response analyses. Using the optimized assay conditions, these compounds were screened against the yeast dependant on either *LmjF*IPCS or AUR1p expression at 11 concentrations between 100 μM to 1.7 nM. From these data, the −logIC_50_ (pIC50) molar unit values of each compound were calculated against each strain and the data plotted (Fig. 2). This allowed analyses of both potency and selectivity, with 500 compounds demonstrating potent activity against *LmjF*IPCS dependant yeast, pIC_50_ ≥ 5 (IC_50_ ≤ 10 μM), and good selectivity when compared to AUR1p dependant yeast, pSI ≥ 1.5 (logSI [pIC_50_ *LmjF*IPCS - pIC_50_ AUR1], where SI is Selectivity Index). This represented a hit rate of 0.0063% for the identification of potent and selective hits against *LmjF*IPCS.

These 500 compounds were subsequently clustered using a sphere exclusion algorithm^30^. Briefly, a fingerprint for each hit was compared to every other molecule in the set and compounds demonstrating significant similarity ( > 0.85 on a scale of 0–1) were sorted into clusters. Two representatives from each cluster plus all singletons, 211 compounds, were taken forward for further analyses.

### Cell-based screening of identified compounds

**Figure 3:**
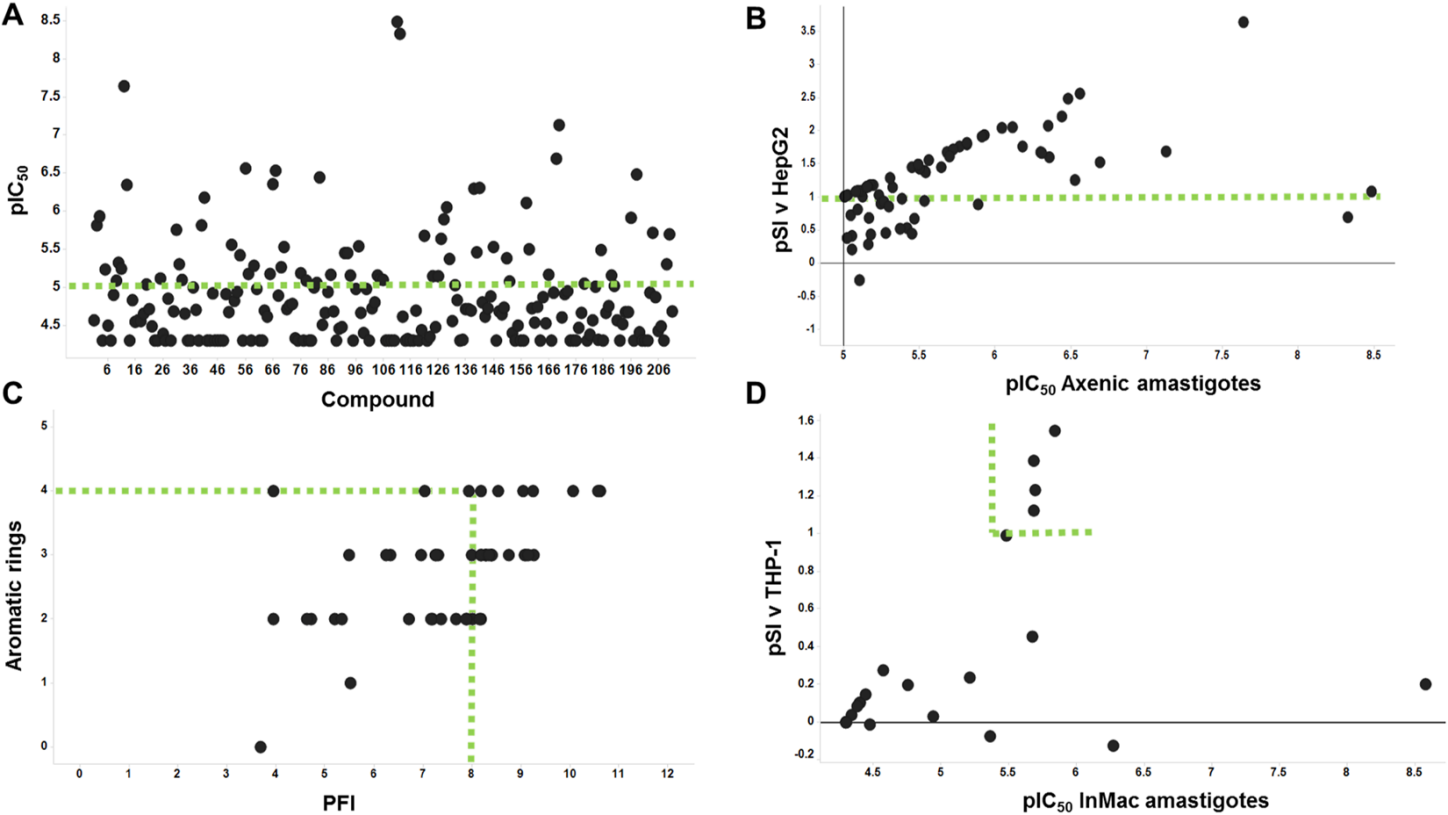
The triage of 211 clustered hits. (**A**) Dose response against axenic *Leishmania donovani* amastigotes, −logIC_50_ M (pIC_50_) ≥5 selected (70 active compounds; on or above green line); (**B**) Comparison of dose response values from (**A**) versus the 70 compounds selectivity (logSI [pSI] where SI is Selectivity Index) against human HepG2 cells, pSI ≥1 selected (49 non-toxic compounds; on or above green line); (**C)** Predicted Property Forecast Index^31^ (PFI) of these compounds against the number of aromatic rings, PFI ≤ 8 and aromatic rings ≤ 4 selected (25 small/hydrophobic compounds; on or within green lines); (**D**) Comparison of dose response values of these 25 compounds for intra-macrophage *Leishmania donovani* amastigotes (InMac) versus the selectivity (pSI) against human THP-1 macrophages, the host, pSI ≥ 1 selected (5 selective compounds; on or within green lines).

To establish the antileishmanial activity of the 211 selected hits, they were screened against *L*. *donovani* axenic amastigotes (the mammalian, pathogenic form) in a dose response assay with 11 compound concentrations between 50 μM and 0.85 nM. The data revealed 70 compounds with a calculated pIC_50_ ≥ 5 (IC_50_ ≤ 10 μM; Fig. 3A; SI Table).

**Figure 4:**
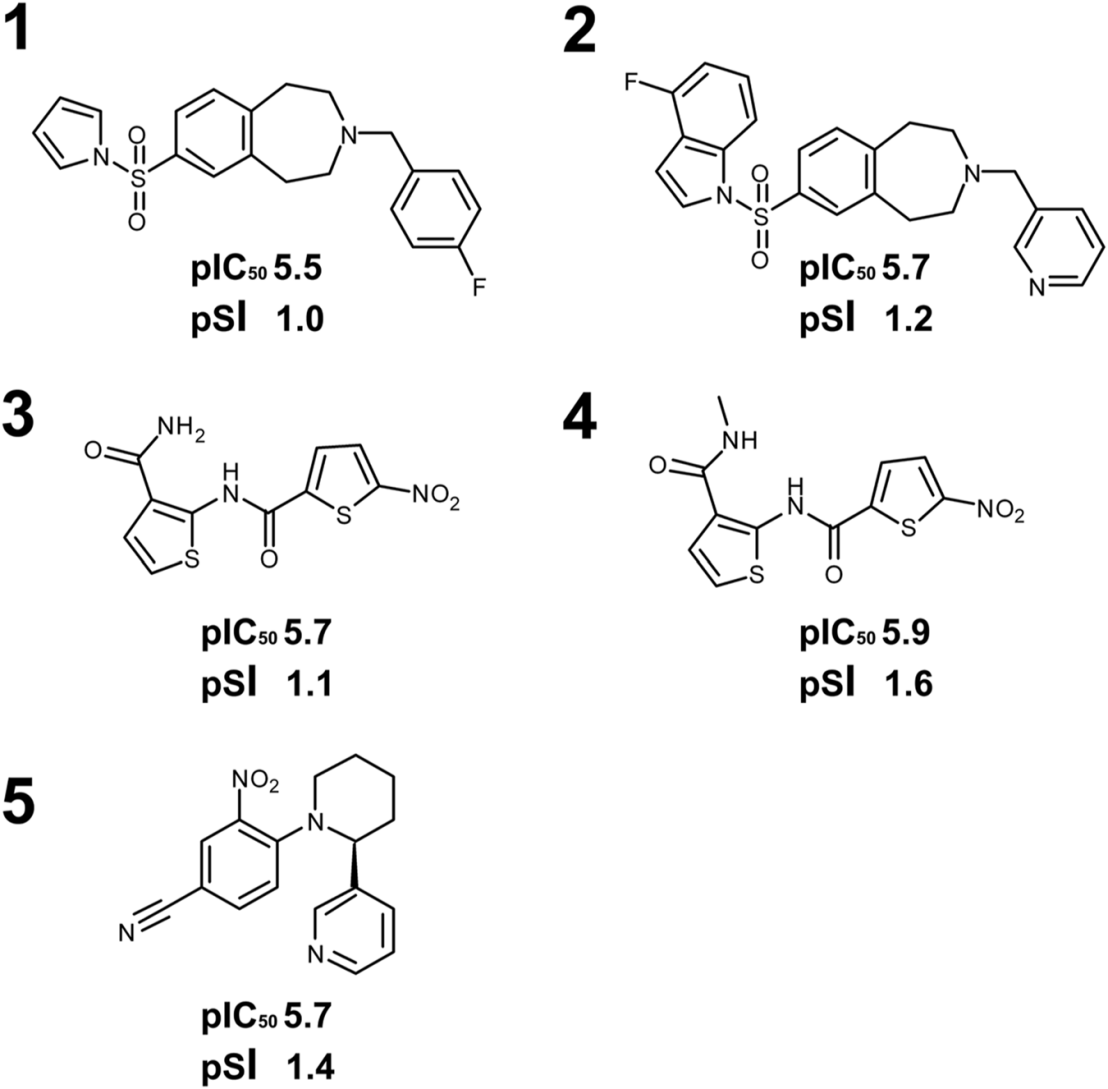
The 5 identified selective, non-toxic, hits. −logIC_50_ M (pIC_50_) and logSI (pSI, where SI is Selectivity Index) values for infected macrophage assay indicated.

Following a screen against HepG2 cells for mammalian cytotoxicity (11 compound concentrations between 100 μM and 1.7 nM), comparing these data with that for the 70 *Leishmania* hits allowed identification of selective antileishmanial compounds with pSI ≥ 1 (SI ≥ 10); 49 compounds fulfilled these criteria (Fig. 3B). Following a further screen based on the predicted physicochemical properties of these 49 compounds, those compounds showing a Property Forecast Index^31^ (PFI) ≤8 and ≤4 aromatic rings were selected (Fig. 3C; SI Table 1)). These 25 compounds have relatively low molecular weights and high levels of hydrophilicity indicating they are likely to be soluble and favourable for development, and therefore were progressed to screening against *L*. *donovani* infected THP-1 macrophages, the truest representation of the pathogenic state available *in vitro*. The 25 compounds were screened in a dose response assay with 11 compound concentrations between 50 μM and 0.85 nM. Given the additional membrane barriers the compounds must cross to reach the intra-macrophage amastigotes, the fact that only 7 (out of 25) were completely ineffective while 10 of the remaining 18 active hits demonstrated activity at pIC_50_ ≥ 5 (IC_50_ ≤ 10 μM) was promising. Of these 10, 4 had a pSI with respect to the activity against THP-1 macrophages of ≥1 (Fig. 3D; SI Table 1), another had a pSI of 0.99 and was also retained. Of these 5 hits (Fig. 4), the benzazepane compounds (**1** and **2**) were both active in the low μM range against intra-macrophage amastigotes, pIC_50_ 5.5 and 5.7 respectively. The dithiophene compounds (**3** and **4**) exhibited similar levels of activity, pIC_50_ 5.7 and 5.9 respectively, however, the presence of the nitro group in this class was a concern given the link with toxicity issues^32^. Similarly, the singleton (**5**) possesses a nitro group. In light of the above, the benzazepanes were taken forward for further analyses in a secondary screening platform^19,22^.

### Verification of the benzazepane mode of action

**Figure 5:**
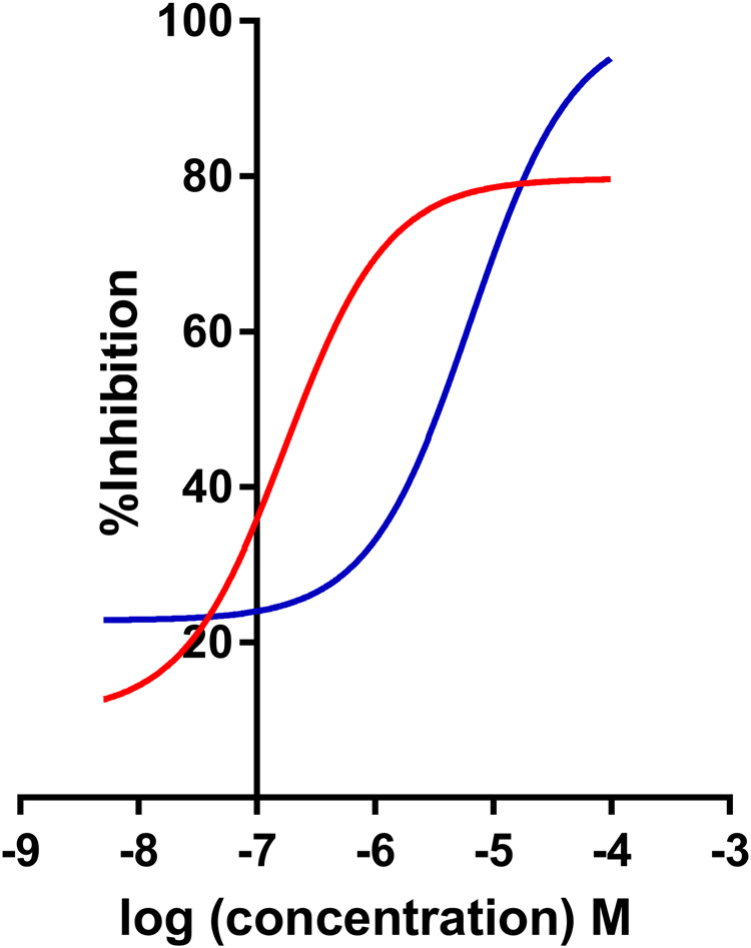
*In vitro* activity against *LmjF*IPCS. Activity of compound **1** (red; −logIC_50_ M [pIC_50_] 6.8; 95% CI: 7.0–6.5) and compound **2** (blue; pIC_50_ 5.2; 95% CI: 5.5–4.9) against *LmjF*IPCS *in vitro*. Values are from at least 3 independent experiments.

Utilizing the availability of an in-house designed and validated biochemical assay for *LmjF*IPCS^19,22^, following re-synthesis in the free-base form (Aptuit, Verona, Italy) the two benzazepane hits were biochemically analyzed in a dose response assay (8 compound concentrations between 100 μM and 46 nM; Fig. 5). Whilst compound **2** was a modest inhibitor of *LmjF*IPCS (pIC_50_ 5.2), compound **1** was a potent, sub μM, inhibitor of the enzyme (pIC_50_ 6.8).

However, whilst compound **1** was antiprotozoal and a potent IPC synthase inhibitor, it was unclear whether the mode of action was on-target. To analyze this further, the in-house availability of a sphingolipid mutant *L*. *major* (*Δlcb2*) was exploited. This line^24^ lacks serine palmitoyltransferase activity, and whilst *LmjF*IPCS is still present it is redundant in the absence of *in situ* substrate. Therefore, specific inhibitors of *LmjF*IPCS should be inactive against *L*. *major Δlcb2*. However, in a dose response assay, compound **1** demonstrated no significant difference in activity against both lines of *L*. *major* (pIC_50_ 5.5 for wild type and *Δlcb2*: SI Fig. 1)). Therefore, it is likely that off-target activity has a role in the antileishmanial activity of this benzazepane.

**Figure 6:**
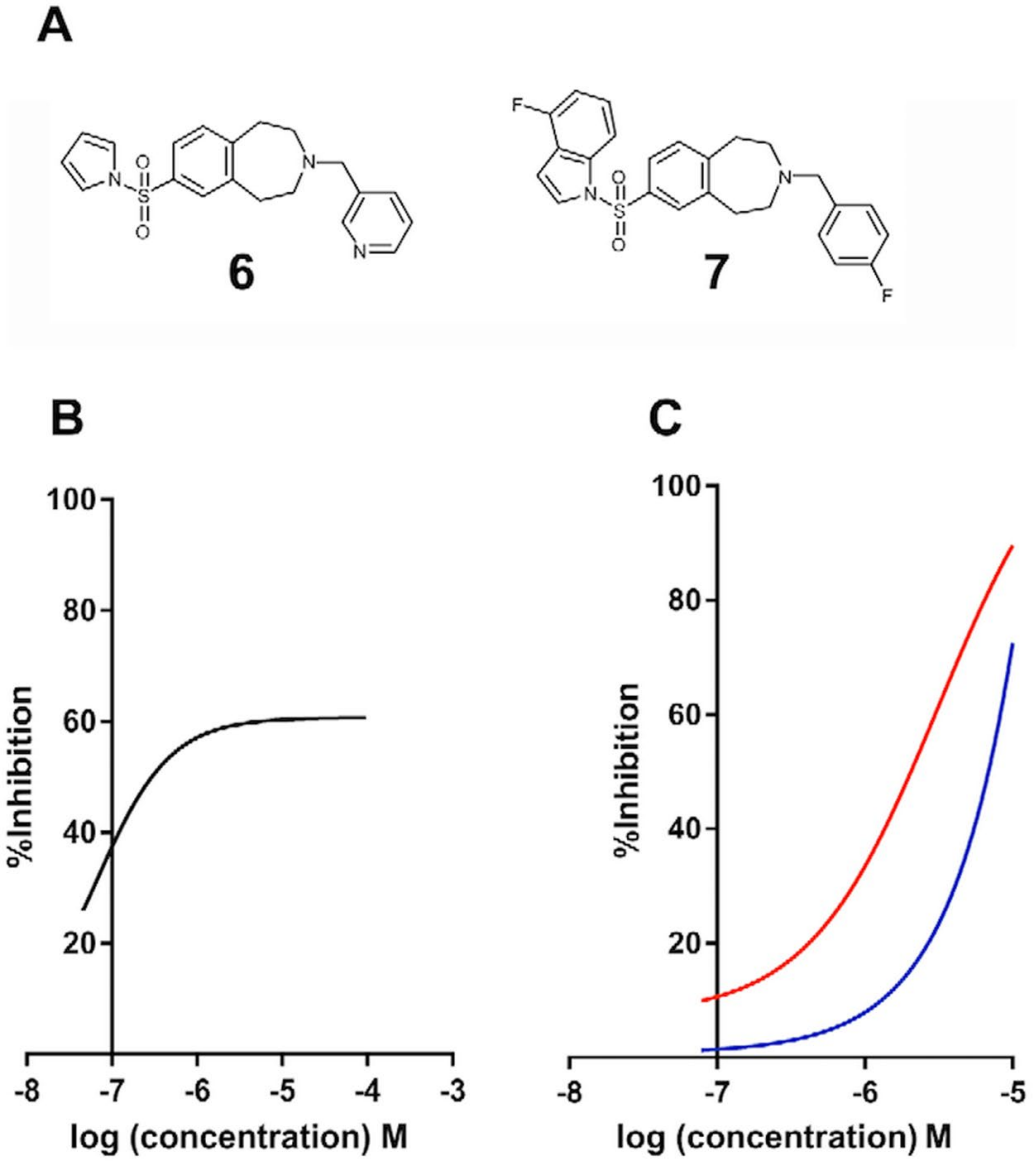
The discovery of a selective inhibitor. (**A**) Synthesized chimeric compounds **6** and **7**; (**B**) activity of compound **7** against *LmjF*IPCS *in vitro* (−logIC_50_ M [pIC_50_] 7.2; 95% CI: 7.8–6.9); (**C)** activity of compound **7** against wild type *Leishmania major* (red; pIC_50_ 5.5; 95% CI: 5.8–5.2) and the mutant, *ΔLCB2* (blue; pIC_50_ < 5). Values are from at least 3 independent experiments.

**Figure 7:**
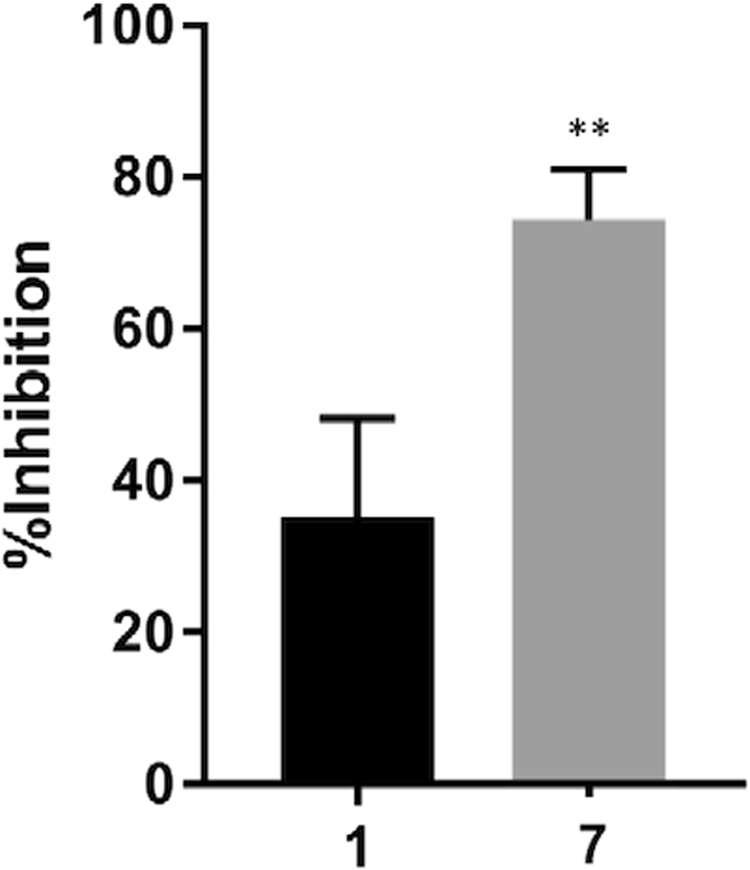
*In cellulo* activity against *LmjF*IPCS. Compound **7** demonstrated a greater activity against *in cellulo LmjF*IPCS than compound **1**. *L*. *major ΔLCB2* treated with 10 μM of compound prior to metabolic labelling with BODIPY FL C_5_-ceramide. Inhibition of the incorporation of label into IPC was measured following HPTLC in comparison with a control (vehicle treated). Values are from 3 independent experiments. P = 0.006 in students’ t-test indicating the difference is significant (**≤ 0.01).

Further synthetic efforts (Aptuit, Verona, Italy) led to the synthesis of compounds bearing the left-hand side of **1** and the right-hand side of **2** (compound **6**) and *vice versa* (compound **7**; Fig. 6A). Compound **6** lost activity against *LmjF*IPCS in the *in vitro* assay; however, compound **7** maintained a pIC_50_ 7.2; although it did not achieve greater than approximately 60% inhibition even at higher concentrations (Fig. 6B). In addition, and importantly, compound **7** demonstrated selectivity for *L*. *major* wild type over the *Δlcb2* mutant (pIC_50_ 5.5 versus <5; Fig. 6C). The selectivity of compound **7** for the wild type parasite over a line with a redundant *LmjF*IPCS (*Δlcb2*), strongly supported a target-directed mode of action. Furthermore, compound **7** demonstrated considerably greater *in cellulo* inhibition of IPC synthase activity than compound **1**, as determined by incorporation of BODIPY^®^ FL C_5_-ceramide into IPC in the presence of the compounds (Fig. 7). Unfortunately, activity against intramacrophage *L*. *donovani* was lost, possibly due to a lack of compound transport to the site of action or intramacrophage stability. However, these data demonstrated that the efficacy of compound **7** against *L*. *major* is on-target inhibition of IPC synthase, thus supporting the status of *LmjF*IPCS as a drug target like its orthologues in the related trypanosomatid pathogen, *T*. *brucei*^20^.

## Discussion

The identification of leads for the discovery of new drugs for the Neglected Tropical Disease leishmaniasis is a recognized priority^2,6^. To achieve this aim the development of strong industry-academia partnerships, bridging chemistry and biology, is vital^23^. Much of such collaborative efforts have employed HTS to identify antiprotozoal compounds, however high content phenotypic screening has produced a poor return of hits against *Leishmania* spp when compared with the related *Trypanosoma* spp^14^. This demonstrated the central importance of target-based screening for the discovery of novel antileishmanial hits which represent new chemical entities with new modes of action.

The *Leishmania* IPC synthase has been previously identified as a putative drug target^18,21^, and more recent work has employed a modelling approach to identify coumarin derivatives as enzyme inhibitors with *in vivo* antileishmanial activity^33,34^. To search for new classes of *Leishmania* IPC synthase inhibitors, in a multidisciplinary public-private partnership, we developed a novel yeast-based assay to screen the 1.8 million entity GSK compound library against the protozoan enzyme. To the best of our knowledge, this work reports the largest yeast-based, target-directed assay undertaken to date against targets from any disease state.

5 non-toxic, antileishmanial compounds were identified (a hit rate of 0.00028%), 2 pairs and one singleton. On the basis of a lack of nitro groups the benzazepanes (compounds **1** and **2**) were selected to take forward. Both of these demonstrated sub μM activity against axenic *L*. *donovani* amastigotes (pIC_50_, 7.6 and 6.4 for compounds **1** and **2** respectively; SI Table), and low μM activity against *L*. *donovani* infected macrophages (pIC_50_, 5.5 and 5.7 for compounds **1** and **2** respectively; SI Table). The activity of these compounds in cell based assays, when compared to other recently predicted *Leishmania* IPC synthase inhibitors^33,34^, was promising. These activity levels were similar to those seen for miltefosine, the only oral therapy for leishmaniasis but with an unclear mode of action^2,35^. However, they were one or 2 orders of magnitude lower than those for amphotericin B, which functions through selective sterol binding^2,35^.

Importantly, both benzazepanes demonstrated activity against the *Leishmania* IPC synthase *in vitro*, with compound **1** particularly potent (pIC_50_ 6.8). Despite these promising findings, this compound also demonstrated off-target activities in the parasite ( SI Fig.1). However, using a simple ‘mix and match’ approach a benzazepane was identified that had enhanced potency against the enzyme *in vitro* (pIC_50_ 7.2) and *in cellulo* (Fig. 7) and demonstrated more selective, on-target antileishmanial activity (Fig. 6).

In summary, this research demonstrated the tractability of yeast-based uHTS to identify inhibitors of challenging, membranous targets such as IPC synthase, which are on-target and specific. Furthermore, the benzazepanes identified represent a new class of antileishmanial compounds with a new mode of action. Further development of these may lead to novel therapies for the Neglected Tropical Disease, leishmaniasis.

## Methods

### Yeast cell assay

The complete open reading frames of *LmjF*IPCS and AUR1p was amplified from genomic DNA using Phusion Flash PCR master mix (ThermoFisher) according to manufacturer’s guidelines. Primers were for T4 ligation of *LmjF*IPCS (F_**BamHI** cgc**ggatcc**ATGACGAGTCACGTGACAGC and R_**HindIII** ccc**aagctt**TTAGTGCTCAGGCAAAGCCGCCG) and In-Fusion cloning (Clontech) of AUR1 (F ctcactatagggcccATGGCAAACC and R tccatgtcgacgcccTTAAGCCCTC). The open reading frames were subsequently cloned into the pESC-LEU vector (Agilent) and verified by sequence analyses, creating pESC-LEU_ *LmjF*IPCS and pESC-LEU_AUR1. In this vector, expression of the open reading frame was under the control of a galactose promoter. pESC-LEU_ *LmjF*IPCS and pESC-LEU_AUR1 were subsequently transformed into *S*. *cerevisiae* strain *α ade*^*−*^.*lys*^*−*^.*leu*^*−*^.*Δaur1*^*−*^.pRS316.*URA*^+^.*ScAUR1*^+^^27^ as previously described^18–20^ and selected on SD -TRP -URA -LEU agar (0·17% Bacto Yeast Nitrogen Base, 0·5% ammonium sulphate, 2% glucose, containing the appropriate nutritional supplements) at 30 °C. Clones were then ‘cured’ of the pRS316-URA plasmid by selection on SGR -TRP –LEU +FOA agar (0·17% Bacto Yeast Nitrogen Base, 0·5% ammonium sulphate, 0.1% galactose, 1% raffinose, 0.1% 5-Fluoroorotic Acid Monohydrate (FOA) containing the appropriate nutritional supplements) at 30 °C, creating *α ade*^*−*^.*lys*^*−*^.*leu*^*−*^.*Δaur1*^*−*^.pESC-LEU_*LmjF*IPCS and *α ade*^*−*^.*lys*^*−*^.*leu*^*−*^.*Δaur1*^*−*^.pESC-LEU_AUR1. Following validation by PCR and propagation in SGR -TRP –LEU, frozen stocks of both yeast lines were created (OD_600_ = 10). 10 µM FDGlu and *α ade*^*−*^.*lys*^*−*^.*leu*^*−*^.*Δaur1*^*−*^.pESC-LEU_*LmjF*IPCS or *α ade*^*−*^.*lys*^*−*^.*leu*^*−*^.*Δaur1*^*−*^.pESC-LEU_AUR1 (defrosted on ice) at OD_600_ = 0.0625 in SGR -TRP -LEU were prepared and dispensed into 1536-well plates (5 µl per well; ThermoFisher) with appropriate concentrations of compound and controls (vehicle or 1 mM cycloheximide). Volumes were dispensed using a Multidrop Combi Reagent Dispenser, a Multidrop Combi nL or a Labcyte Echo^®^ liquid handler (ThermoFisher). Plates were then incubated, in duplicate, at 30 °C for 25 hours prior to the addition of phosphate buffer (0.25 M, pH 7.0, 5 µl per well) and fluorescence was read at Ex480/Em540 using a ViewLux ultraHTS Microplate Imager (PerkinElmer). Data were analyzed using GSK-developed Statistical Online Data Analysis Software (SODA) and ActivityBase (IDBS).

### *Leishmania* axenic cell assays

As previously described^36^, *L*. *donovani* (MHOM/SD/62/1S-CL2D, LdBOB) axenic amastigotes in growth medium (6 µl) were added to a final concentration of 5.0 × 10^3^ ml^−1^ with compounds and controls (vehicle and assay media alone, with the robustness of the platform indicated by Z’ values > 0.5)^29^ in 1536-well plates (Greiner Bio-One FLUOTRAC). The plates, in at least triplicate, were incubated at 37 °C for 72 hours under 5% CO_2_. Resazurin solution was added, the plates were incubated at room temperature for 4 hours and then the fluorescence read at Ex528/Em590, using an EnVision Multilabel plate reader (PerkinElmer). Volumes were dispensed and data analyzed as above.

From a previously described protocol^22^, *L*. *major* (MHOM/IL/81/Friedlin, wild type and ΔLCB2 mutant)^24^ were added to 96-well plates (Corning) at 1 × 10^4^ ml^−1^ in growth media with appropriate concentrations of compounds and controls (vehicle and assay media alone, with the robustness of the platform indicated by Z’ values > 0.5)^29^ in 100 µl. Following incubation at 26 °C for 72 hours, alamarBlue (ThermoFisher) was added, the plates were incubated at 26 °C for 4 or 8 hours and fluorescence was read at Ex540/Em600, using a FLx800 Fluorescence Microplate Reader and Gen5 1.08 Data Analysis Software (BioTek). Analyses were carried out using GraphPad Prism 7.

### Mammalian cell assay

HepG2 cells in media were added (25 µl) at final concentration of 1.2 × 10^5^ ml^−1^ to 384-well plates (Greiner Bio-One FLUOTRAC) and incubated with compounds and controls (vehicle and cycloheximide) at 37 °C for 48 hours under 5% CO_2_ and at 80% relative humidity. The plates, in at least duplicate, were incubated at room temperature for 30 minutes prior to the addition of CellTiter-Glo (Promega), followed by incubation at room temperature for 10 minutes and measurement of the luminescent output using a ViewLux ultraHTS Microplate Imager (PerkinElmer). Volumes were dispensed using a Multidrop Combi Reagent Dispenser, a Multidrop Combi nL or a Labcyte Echo liquid handler (ThermoFisher). Data were analyzed using GSK-developed Statistical Online Data Analysis Software (SODA) and ActivityBase (IDBS).

### Intramacrophage *Leishmania donovani* assay

This protocol was based on that previously described^36^. In brief, THP-1 (human monocytic leukemia) at 6 × 10^5^ ml^−1^ were infected with eGFP expressing *L*. *donovani* LdBOB amastigotes at 6.0 × 10^6^ ml^−1^ and incubated overnight at 37 °C. Following washing, infected cells were trypsinized and harvested before dispensation into a sterile 384-well plate, at 6.0 × 10^4^ ml^−1^ in 50 µl post-differentiation growth medium with compounds and controls (vehicle and amphotericin B, with the robustness of the platform indicated by Z’ values > 0.5)^29^. The plates, in at least duplicate, were incubated at 37 °C for 96 hours under 5% CO_2_. After fixation with 4% (v/v) formaldehyde in PBS at room temperature for 30 minutes and washing, cells were stained with DAPI (Sigma-Aldrich) at room temperature for 30 minutes, washed, and imaged at Ex405/Em460 and Ex488/Em509 using the Opera^®^ High Content Screening System (PerkinElmer). Volumes were dispensed as above. Images were analyzed using the automated Acapella High Content Imaging and Analysis Software (PerkinElmer).

### Biochemical assay

Microsomal material was prepared and CHAPS washed from *α ade*^*−*^.*lys*^*−*^.*leu*^*−*^.*Δaur1*^*−*^.pESC-LEU_ *LmjF*IPCS as previously described^19,22^. Briefly, each compound at the desired concentration was incubated, in at least triplicate, in 96-well plates (Corning) in phosphate buffer (71.4 mM, pH 7.0) with PI (100 µM, final concentration, Avanti Polar Lipids), CHAPS (600 µM, Sigma-Aldrich) and CHAPS-washed microsomal membranes (0.6 U enzyme per reaction) for 15 minutes at 30 °C. NBD-C_6_-ceramide (5 µM; ThermoFisher) was added, final volume of 40 µl per well, and the plates incubated at 30 °C for a further 25 minutes. Following quenching with 200 µl methanol per well, the reaction product was separated using exchange chromatography in 96-well filter plates (Millipore)^19,22^ and the fluorescence measured at Ex460/Em540 using a FLx800 Fluorescence Microplate Reader and Gen5 1.08 Data Analysis Software (BioTek). Analyses were carried out using GraphPad Prism 7.

### *In cellulo* IPC synthase assay

The *L*. *major Δlcb2* mutant^24^, maintained in Schneider’s media (Sigma-Aldrich) with 15% sera (ThermoFisher), were washed and 200 µl, at 1 × 10^7^ ml^−1^, incubated in serum free media for 60 minutes at 26 °C before the addition of 10 µM of the compounds, in at least triplicate, and further incubation for 18 hours. Subsequently, the reaction was initiated by the addition of BODIPY FL C_5_-ceramide complexed to BSA (1.25 µl of 0.5 mM, ThermoFisher). Following further incubation at 26 °C for 1 hour, the lipids were extracted by phase separation in chloroform:methanol:water (10:10:3, 200 µl) and fractionated on HTPLC plates, as previously^18^. The fluorescence was read at Ex475/Em520 using a Fuji FLA-3000 plate reader and AIDA Image Analyser software (version 3.52).

## Electronic supplementary material

Supplementary Information

Supplementary Table
