# Supplementary material for: Identifying inhibitors of the Leishmania inositol phosphorylceramide synthase with antiprotozoal activity using a yeast-based assay and ultra-high throughput screening platform
Source: Sci Rep. 2018 Mar 2;8:3938. doi: 10.1038/s41598-018-22063-9 (PMC5834442; doi:10.1038/s41598-018-22063-9)

Identifying inhibitors of the *Leishmania* inositol phosphorylceramide synthase with antiprotozoal activity using a yeast-based assay and ultra-high throughput screening platform

Jennifer L. Norcliffe, John G. Mina, Emilio Alvarez, Juan Cantizani, Francisco de Dios-Anton, Gonzalo Colmenarejo, Silva Gonzalez-Del Valle, Maria Marco, José M. Fiandor, Julio J. Martin, P.G. Steel and P.W. Denny

Supplementary Information

SI Table 1

Data from analyses of the clustered 211 hits. Structure – chemical structure (where available); Cmpd Number – compound reference number; PFI - Property Forecast Index; A'ring – number of aromatics ring; pIC50 LmjIPCS – -logIC_{50} mol against *α ade*$^-$.*lys*$^-$.*leu*$^-$.*Δaur1*$^-$.pESC-LEU_*Lmj*IPCS; pIC50 AUR1 – -logIC_{50} mol against *α ade*$^-$.*lys*$^-$.*leu*$^-$.*Δaur1*$^-$.pESC-LEU_AUR1; pIC50 axenic – -logIC_{50} mol against *L. donovani* (MHOM/SD/62/1S-CL2D, LdBOB) axenic amastigotes; pIC50 axenic $\geq$5 **TRUE** or **FALSE**; pIC50 HepG2 – -logIC_{50} mol against HepG2 cells; pSI v HepG2 – logSI (Selectivity Index) of compounds for axenic amastigotes over HepG2 cells; pSI v HepG2$\geq$1, PFI$\leq$8, A'ring$\leq$4 **TRUE** or **FALSE**; pIC50 InMac – -logIC_{50} mol against intramacrophage *L. donovani*; pIC50 THP-1 – -logIC_{50} mol against THP-1 macrophages; pSI v THP-1 - logSI (Selectivity Index) of compounds for axenic amastigotes over THP-1 macrophages; InMac $\geq$5, pSI v THP1 $\geq$ 1 **TRUE** or **FALSE**; Hit Ref – Hit reference number in article

pIC50 of 4 (4.3 for iMac and THP-1 assay) are the minimum values obtainable from the assays, therefore actual pSI values could be higher.

SI Figure 1

The activity of compound **1** against wild type *Leishmania major* (red; -logIC_{50} mol [pIC_{50}] 5.5; 95% CI: 5.9-5.0) and the mutant, *Δlcb2* (blue; pIC_{50} 5.5; 95% CI: 5.7-5.2). Values are from 3 independent experiments.

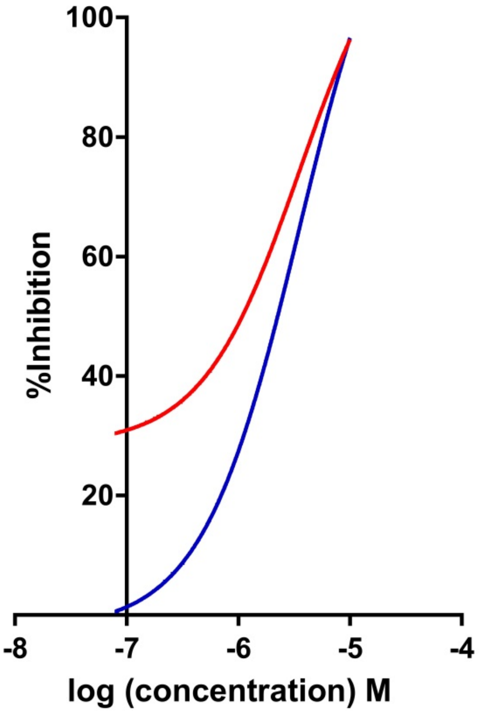

Supplement: Supplementary file 1 — Supplementary Information [file 41598_2018_22063_MOESM1_ESM.pdf]
